# Yōhei Sasakawa: a lifetime fighting leprosy

**DOI:** 10.2471/BLT.22.030922

**Published:** 2022-09-01

## Abstract

Yōhei Sasakawa speaks with Vijay Shankar Balakrishnan about his work tackling leprosy and the discrimination associated with it.

*Q: In the public health community you are perhaps best known for your philanthropic work on leprosy. How did you come to that focus?*

A: My father was a huge influence. I did not live with him until I was 16 years old, but I have vivid memories of sitting at the dinner table, listening to his conversations with the guests, many of whom came to him for help with their problems. His interest in leprosy was sparked by the disappearance of a young woman from his life, who – he later discovered – had been diagnosed with leprosy. The experience troubled him deeply and he made leprosy elimination his life's mission. I later had some experiences that confirmed me in my resolve to follow him on that path.

*Q: Can you talk about those experiences?*

A: My father was chairman of the Japanese Veterans and Soldiers Association and used to go abroad as part of his duties. He also used to go to leprosy sanatoria. I accompanied him on those trips, but I did not dare to enter the sanatoria until in 1965, at the age of 26, I decided to visit one in South Korea. It was a life-changing experience for me. I saw the terrible burden of illness that people had to carry as well as the discrimination and mistreatment they suffered. And I saw the way my father was with them, getting so close, putting his arms around them to console them. That experience really left a mark on me and, even though I did not work on leprosy immediately, having other responsibilities, the seed was planted. My father had already set up the Nippon Foundation by that time, using Japanese motorboat racing revenue to fund global maritime development and philanthropic activities, and in 1974 he co-founded the Sasakawa Health Foundation which was set up with the aim of eradicating leprosy worldwide. 

*Q: How did a business entrepreneur go about tackling this challenging public health issue?*

A: By reaching out to leading Japanese thinkers in the field, including researchers such as Professor Morizo Ishidate, co-founder of Sasakawa Health Foundation (SHF), who was later joined by Dr Yo Yuasa as medical director to work on the elimination of leprosy, notably by supporting efforts to develop new treatments. Professor Ishidate was the first Japanese national to synthesize promin, a drug that was originally intended to treat malaria and tuberculosis, but which proved effective against leprosy. Of course, it was not enough to come up with new treatments. It was also necessary to find ways to engage with health authorities worldwide to find the most effective ways to deliver them. Professor Ishidate firmly believed that leprosy elimination could only be achieved by partnering with the health ministries in each endemic country and my father initially considered funding them directly. However, Professor Kenzo Kiikuni, one of the founding members of SHF, persuaded my father to donate US$ 1 million to the World Health Organization (WHO), arguing that WHO was better placed to ensure optimal use of the funds. Dr Halfdan Mahler, the then Director-General, gratefully accepted the donation but requested that my father spend the money on smallpox eradication, a major priority for the Organization at that time. My father agreed, and that was the beginning of SHF’s collaboration with WHO.

“Leprosy is still a forgotten and […] invisible disease.”

Of course, SHF continued working on leprosy, promoting science-based, internationally coordinated solutions, collaborating with health ministries, and providing human resources and medicines. These included the provision of multi drug therapy (MDT) comprised of rifampicin, clofazimine and dapsone, which became the standard treatment for the disease in the mid-1980s. The Nippon Foundation funded the provision of the drugs free of charge to governments and health authorities around the world between 1995 and 1999, after which the pharmaceutical company Novartis ensured the supply. The SHF also contributed to ensuring optimal delivery of the drugs procured. 

One of the many challenges faced in getting MDT to the populations who needed it was to deliver high-quality leprosy drugs to primary health centres in rural areas. Dr Hiroshi Nakajima, WHO Director-General at the time, and Dr Yo Yuasa, the Sasakawa Health Foundation's medical director, jointly decided that it would be best to supply them in the form of blister packs. Since that decision was taken, I have personally visited many countries to make sure that blister packs were actually being distributed across those rural areas and have brought the situation to the attention of heads of state or ministers. Such efforts have helped drive the leprosy prevalence rate below 1 case per 10 000 of population (the level at which leprosy is declared to have been eliminated as a public health problem) in numerous countries. In India, for example, prevalence went from 58 per 10 000 in 1983 to 1 per 10 000 in 2005. Timor-Leste became the last country in Asia to do away with leprosy as a public health problem in 2011. 

*Q: You are known for your personal engagement in the field. Why is that so important to you?*

A: First, because it is a demonstration of commitment, but it also provides invaluable opportunities for learning and the exchange of ideas. I have learnt a great deal about the challenges faced on my travels, particularly in regard to discrimination, often supported by discriminatory legislation, and widespread disregard for the populations affected. In some cases this disregard translates into complete ignorance about the disease and the burden it imposes. The sad truth is that leprosy is still a forgotten and to some extent invisible disease in many countries. An important part of my work is to make sure it is visible and to remind people in positions of power that health is a human right, which makes leprosy a human rights issue. In July 2003 I went to the Office of the United Nations High Commissioner for Human Rights to advocate for the leprosy issue being raised before the then United Nations Commission on Human Rights. 

*Q: How can a disease characterized by such devastating symptoms remain invisible?*

A: For a couple of reasons. First, because leprosy does not initially result in a fever or cause other disability, people typically ignore the symptoms at first. Second, because there is tremendous fear of discrimination – of being ostracized, of losing homes, jobs, livelihoods etc. – people living with leprosy tend to hide their symptoms, waiting for two or three years before going to the clinic or hospital, despite the fact that early diagnosis is not difficult and can be done at home. This is one of the reasons we often see more advanced or aggravated cases with their associated disfigurement. This is another reason discrimination is such a central concern, which is why I called on the Japanese government to submit a resolution to the UN General Assembly in 2010. Resolution 65/215 on elimination of discrimination against persons affected by leprosy and their family members was adopted unanimously by all 192 Member States. That resolution has been tremendously helpful, allowing the SHF and other organizations’ representatives to challenge government officials and heads of municipalities on the topic and to help them eliminate the discrimination and stigmatization that exists. We are still working on different approaches to advance this agenda, while also encouraging greater awareness and early diagnosis of the disease.

“There is tremendous fear of discrimination.”

*Q: Can you talk about that work?*

A: The first symptom of leprosy is typically the appearance of light-coloured patches on the body. This is something that we can advise the family members to check themselves to see. If they do see it, a simple diagnostic procedure is to just use the tip of a pencil to touch the skin of those patches. If the person does not feel anything, it is likely that the patches are an early symptom of leprosy. At that point the person should go to a clinic and get tested and start taking MDT. In that way the disease can be stopped before progressing into disfigurement and disability. In some cases, we have encouraged schools to give children a sheet of paper with an illustration of the human body. The children then take this home, to have their parents check them for skin patches, and then mark those on the paper. Where patches are found the school can have the children go to the nearest clinic for medical diagnosis and treatment. Because of discrimination, such efforts are not enough on their own and active case finding is crucial. We have been supporting endemic countries trying to establish a system in which a health worker visits each household to explain the nature of leprosy and create awareness. There are many examples of this kind of work in the field, including the ASHA (Accredited Social Health Activist) initiative whereby community health workers visit each household to proactively find people with leprosy or to dispense MDT, asking the leprosy-positive patients to take the medication in front of them. I would like to see such community health worker systems established in all countries. 

*Q: The Sasakawa Health Foundation's* *work goes far beyond leprosy elimination. Could you talk about some ongoing projects?*

A: (laughing) We would need a longer interview! We are currently trying to address various challenges in Japan, focusing our resources where the government, for administrative or other reasons, is constrained. Our hope is that, once we start, the government will catch up with us to take over or support our activities. One of the projects relates to end-of-life care. A recent survey revealed that about 70% of the elderly would prefer to receive palliative care at home. So, we are trying to create a network of nurses willing to provide such care for people who want to die peacefully at home instead of in a hospital. We are also working on a project to assist people with disabilities to find their place in the workforce and to create an inclusive work environment. We are also working to promote projects to clean up plastic waste in our oceans.

*Q: You sound very busy. Does all that work leave any time for personal projects?*

A: Absolutely. I have just completed another book, about my role as WHO Goodwill Ambassador and efforts to eliminate leprosy and its associated discrimination. I am also in training to climb Mount Fuji. I was inspired by a friend who displayed a flag for our “Don’t Forget Leprosy” campaign at the summit of Mount Everest. I want to do the same on top of Mount Fuji!

